# UV-Initiated Polymerization of Cationic Polyacrylamide: Synthesis, Characterization, and Sludge Dewatering Performance

**DOI:** 10.1155/2013/937937

**Published:** 2013-12-24

**Authors:** Huaili Zheng, Yongjun Sun, Xiaomin Tang, Mingzhuo Tan, Jiangya Ma, Wei Chen, Yong Liao

**Affiliations:** ^1^Key laboratory of the Three Gorges Reservoir Region's Eco-Environment, State Ministry of Education, Chongqing University, Chongqing 400045, China; ^2^GuangDong Wealth Environmental Protection Co., Ltd., Guangdong 529000, China

## Abstract

P(AM-DAC-BA) was synthesized through copolymerization of acrylamide (AM), acryloyloxyethyl trimethyl ammonium chloride (DAC), and butylacrylate (BA) under ultraviolet (UV) initiation using response surface methodology (RSM). The influences of light intensity, illumination time, and photoinitiator concentration on the intrinsic viscosity [*η*] of P(AM-DAC-BA) were investigated. RSM model based on the influencing data was established for optimizing synthetic conditions. It was found that, at light intensity 1491.67 **μ**w*·*cm^−2^, illumination time 117.89 min, and photoinitiator concentration 0.60‰, there was a better material performance achieved. Thus P(AM-DAC-BA) prepared under the above conditions showed excellent dewatering performance that, with 40 mg*·*L^−1^ P(AM-DAC-BA) at pH 7, the residual turbidity of supernatant and the dry solid content were up to 38 NTU, 28.5%, respectively.

## 1. Introduction

In recent years, the mount of sewage treated by municipal wastewater treatment plant increases rapidly, resulting in a large amount of excess activated sludge generated, whose moisture content is up to 99.5%, and the sludge containing organic matter and noxious substances will harm the environment and must be handled properly [1]. Because waste activated sludge is a colloidal system in which small sludge particles form a stable suspension in water and are very difficult to be separated from the water phase, the activated sludge must be pretreated before its mechanical dewatering. To improve the sludge dewatering, flocculants are usually added before dewatering, which contact with sludge particles through flocculation process to change the surface charge of sludge particles, reducing electrostatic repulsion between sludge particles to flocculate and aggregate into large flocs. Floccultaion-coagulation process is a very effective way to improve its dewatering performance and achieve the purposes of sludge dewatering.

Currently sludge conditioners used by municipal wastewater treatment plant are cationic polyacrylamides (CPAMs) [2]. CPAMs have many advantages including low dosage, small amount of sludge produced after flocculation, and being less susceptible to both salt and pH. Flocculation-coagulation process could increase the floc settling velocity flocculated by CPAM and make sludge easy to dewater [3]. CPAMs are usually initiated by heat, microwave, rays, and ultraviolet (UV) [4]. UV-initiated polymerization is efficient, energy-saving, and friendly to environment.

Response surface methodology (RSM) is used to optimize the testing program or the relationship between indicators and factors in mathematical model, using multiple regression equation to fit the second factor and the functional relationship between indicators, through the regression equation analysis to find the optimal process parameters [5, 6]. Box-Behnken design was used to optimize the synthesis conditons [7, 8]. The appliation of Box-Behnken method in wastewater treatment had received a lot of attention, but its application in optimizing the synthesis process of flocculants was rarely studied.

In order to enhance the dewaterability of the waste-activated sludge, P(AM-DAC-BA) in this study was synthesized through UV-initiated polymerization with AM, DAC, and BA as monomers.

To obtain an optimum synthetic condition, the effect of photoinitiator concentration, incident light intensity, and illumination time on intrinsic viscosity was investigated, and using these investigated data, the response surface methodology (RSM) was fitted to analyze their mutual effect and attain a better condition for preparation of P(AM-DAC-BA). The P(AM-DAC-BA) used in sludge dewatering showed that the dewaterability of waste activated sludge was affected by flocculant dosage and pH significantly.

## 2. Experimental

### 2.1. Experimental Material and Instruments

The acrylamide (AM) (Chongqing Lanjie Tap Water Company, Chongqing, China), butyl acrylate (BA) (Tianjin Guangfu Fine Chemical Research Institute, Tianjin, China), acryloyloxye-thyltrimethyl ammonium chloride (DAC, 80% in water) (Guangchuangjing IMP. EXP. Co., Ltd., Shanghai, China), and the photoinitiator 2,2′-azobis(2-methylpropionamide) dihydrochloride (V-50) (Ruihong biological technology, Shanghai, China) were purchased. All the reagents used in this experiment were without further purification. Commercially available cationic polyacrylamide (CPAM) was sourced from Shandong Polymeibio-Chemicals Co., Ltd., China.

Some instruments, such as UV-A ultraviolet ray intensity meter (Beijing Normal University Photoelectric Instrument Factory, China), TU-1901 double beam ultraviolet and visible spectrophotometer (Beijing Purkinje General Instrument Co., Ltd., China), 550Series II infrared spectrometer (BRUKER 4 Company, Switzerland), were used in this study.

### 2.2. Synthesis of P(AM-DAC-BA)

A predetermined mass ratio of AM, DAC, and BA was added into the quartz jar, then adding a certain amount of deionized water, surfactant, and urea. The reagent was mixed and stirred, and then the reaction solution was bubbled with nitrogen gas for 30 min at room temperature, and, subsequently, the predetermined photoinitiator dosage was added. The reaction vessel was exposed to radiation for 120 min with a 500 W high pressure mercury lamp at ambient temperature. After radiation, the product was purified with acetone and ethanol and then was dried and made into powder. Based on the results of the optimized design by response surface method (RSM), the ranges of synthesis condition were listed in Table 2.

### 2.3. Determination of Intrinsic Viscosity [*η*]

The intrinsic viscosity of CPAM was determined by one point method; the detailed method and operating steps were shown in “the determination method of intrinsic viscosity of polyacrylamide” (GB-T12005.1-1989).

### 2.4. Sludge Dewatering Experiments

Experimental sludge was collected from sludge storage tank of Chongqing Baihan wastewater treatment plant; the water content of which was 98.56%, pH value was 7.56–7.62, and the sludge with fine particles was brown and odor. The density of sludge was 1.02 g/mL.

In sludge dewatering experiments, the P(AM-DAC-BA) was added into a 250 mL beaker with 200 mL sludge included. The pH of the sludge was adjusted with 0.1 mol/L NaOH and 0.1 mol/L HCl. Subsequently, it was rapidly mixed by stirring at 120 rpm for 30 s, followed by a 10 min quiescent settling time. Then, the supernatant was extracted from the beaker 1 cm below the solution surface by a syringe for turbidity measurement. Sludge samples were filtered through a vacuum suction filter machine at the pressure of 0.05 MPa. The dry solid content (DS) was evaluated by the following equation:

(1) $$\begin{array}{c} \text{DS}=\frac{W_{1}}{W_{2}}\times 100\%, \end{array}$$

where *W*_1_ (g) is the weight of filter cake at the end of filtration and *W*_2_ (g) is the weight of filter cake after drying at 105°C for 24 h.

## 3. Results and Discussion

### 3.1. Experiments Design and Results of Regression Model Equation

In order to optimize synthetic condition, establish the model for UV-induced polymerization of P(AM-DAC-BA) and investigate the UV effect on [*η*] of P(AM-DAC-BA); three significant levels of ultraviolet factors and [*η*], which had a strong impact on dewaterability efficiency of waste sludge, were selected for the statistical method, where Box-Behnken experimental design was used to investigate the mutual influence among the impact factors on intrinsic viscosity of P(AM-DAC-BA) [9, 10]. The factor levels selected, as shown in Table 1, indicated that the upper level corresponds to +1, the lower level to −1, and the basic level to zero. The experimental result was analyzed using professional software, Design-Expert V8.0.6.

On the basis of response surface methodology, Box-Behnken experimental designs were selected for optimizing and modeling of the preparation of P(AM-DAC-BA), with *X*_1_, *X*_2_, and *X*_3_ representing photoinitiator concentration, incident light intensity, and illumination time, respectively. Experimental ranges and levels of the independent test variables were shown in Table 2. Seventeen observed responses were used to calculate the model using Box-Behnken method. From the experimental data, the quadratic regression models were established using Design Expert V8.0.6, as shown in

(2) [η]=1.86+0.11X1+0.38X2+0.34X3+0.075X1X2+0.14X1X3−0.068X2X3−0.27X12−0.15X22−0.32X32.

The adequacy of the models was investigated using the analysis of variance (ANOVA) and the results were shown in Table 3. *F*-value of the model was 37.33 and the *P* value was less than 0.0001, indicating that the model was significant. In addition, the value of the determination coefficient, *R* ^2^ = 0.9796, indicated that the sample variation was 97.96% for [*η*], which was attributed to the independent variables, and only 2.04% of the total variations were not explained by the model, suggesting a strong correlation between the predicted and the observed values. Furthermore, the analytical result indicated a better precision and reliability of the experiments being carried out. From the above investigation, it showed that the model was adequate for the prediction of intrinsic viscosity.

### 3.2. Mutual Effect of Parameters

**Figure 1:**
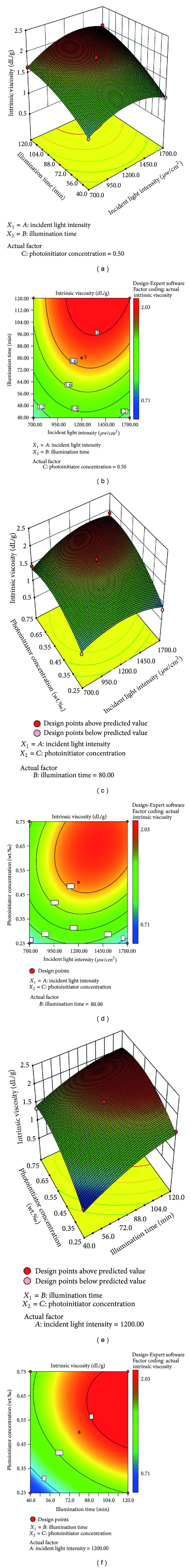
Mutual effect of photoinitiator concentration, incident light intensity, and illumination time on intrinsic viscosity [*η*].

In order to investigate the various factors and their mutual effect on the intrinsic viscosity of P(AM-DAC-BA), the response surface plots were used to display the response as a function of two factors by keeping the third factor constant, and two-dimensional response surface contours were plotted to investigate the mutual effect of operational variables. The response variables were shown in Figure 1.

Figures 1(a) and 1(b) show the intrinsic viscosity as a function of incident light intensity and illumination time, Figures 1(c) and 1(d) show the intrinsic viscosity as a function of incident light intensity and photoinitiator concentration, and Figures 1(e) and 1(f) show the intrinsic viscosity as a function of illumination time and photoinitiator concentration. Figures 1(a)–1(f) demonstrate the mutual effect of photoinitiator concentration, incident light intensity, and illumination time on the intrinsic viscosity. It indicated that the increase in photoinitiator concentration and illumination time might lead to the increase of the intrinsic viscosity, especially under the condition of higher photoinitiator concentration and longer illumination time. There was a maximum value in the intrinsic viscosity as the incident light intensity increased within the investigated range of incident light intensity.

### 3.3. Model Validation

In order to obtain the maximum value of the intrinsic viscosity, the response optimizer in the Design Expert V8.0.6 software was used to search for an optimal synthetic condition. The best condition for the intrinsic viscosity of P(AM-DAC-BA) in theory was that photoinitiator concentration was 0.60‰, incident light intensity 1491.67 *μ*w/cm^2^, and illumination time 117.89 min, respectively. The predicted results were calculated by the Design Expert V8.0.6 software based on (2). In order to verify the prediction results, the parallel experiments were carried out. The chosen conditions for cationic degree, photoinitiator concentration, incident light intensity, and illumination time are all listed in Table 4, along with the predicted and measured results. As shown in Table 4, the measured intrinsic viscosity was close to the predicted values using the regression model, indicating the suitability of the fitted polynomial models with respect to the experimental data. But this model was just a theoretical model, it could give a good prediction including optimal conditions and general condition.

### 3.4. FTIR Spectrum

**Figure 2:**
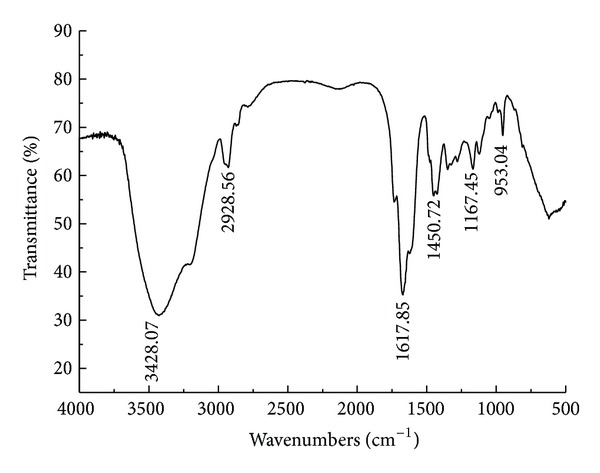
FTIR spectrum of P(AM-DMDAAC-BA).

Figure 2 shows the FTIR spectrum of P (AM-DMDAAC-BA). The absorption bands observed at 3428.07 cm^−1^ and 1167.45 cm^−1^ were originated from strong stretching vibration of amino group and carbonyl group of amide in the AM chain, respectively. The bending vibration absorption peaks of –CH_2_–N^+^(CH_3_)_3_ group of DAC appeared at 1450.72 cm^−1^. The stretching vibration absorption peak at 1671.85 cm^−1^ was attributed to –C=O group in the AM and BA [11]. The analytical results of the FTIR spectrum conformed the formation of the terpolymer of AM, DAC, and BA.

## 4. Sludge Dewatering Tests

### 4.1. Effect of Dosage on Sludge Dewatering Performance

**Figure 3:**
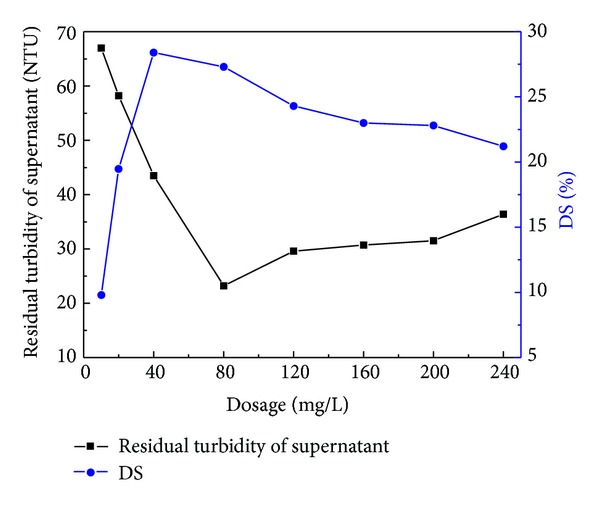
Effect of dosage on sludge dewatering performance.

The effect of dosage on the sludge dewatering performance is shown in Figure 3. The residual turbidity decreased with the increase of the flocculant dosage, which reached a minimum at flocculant dosage of 80 mg·L^−1^. When the dosage was higher than 80 mg·L^−1^, the residual turbidity increased. With increasing dosage, the dry solid content distinctly decreased firstly and then increased gradually. When the dosage was 40 mg·L^−1^, the lowest DS of 28.4% was obtained.

When the dosage was low, the flocculant could not neutralize negatively charged particles, and the sludge flocs were small and friable, not sufficient to improve sludge dewatering performance. So the supernatant turbidity was high and DS was low. When the dosage was too high, the flocculant caused flocculation system positively charged, increasing repulsion between flocs and leading to the restabilization of the flocs [12, 13]. Excessive dosage would increase the viscosity of the water in sludge, making it not easy to filter. Finally, the dry solid content of sludge cake was reduced [14]. Therefore, an appropriate flocculant dosage of 40 mg·L^−1^ for dewatering of the waste activated sludge was necessary.

### 4.2. Effect of pH on Sludge Dewatering Performance

**Figure 4:**
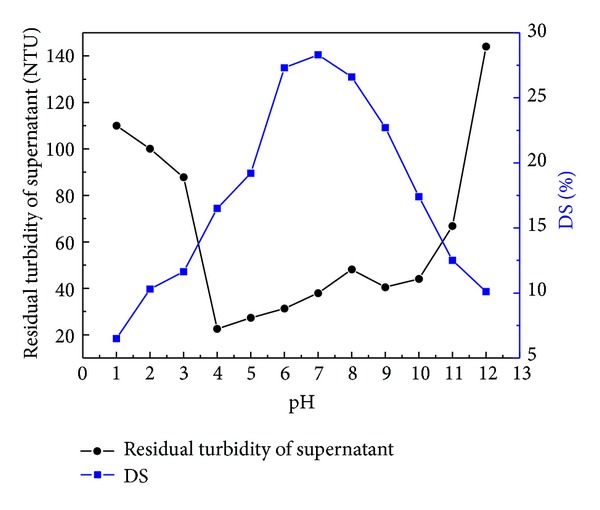
Effect of pH on sludge dewatering performance.

Figure 4 shows the effect of pH on the sludge dewatering performance. With the increase of the pH, the residual turbidity of the supernatant decreased significantly, but increasing pH further would cause an increase. In contrary, as increasing the pH the dry solid content increased and an obvious decrease was found in higher pH value. The experimental results showed that the pH between 6 and 8 favored the sludge dewatering where the dry solid content of the filter cake did not change largely.

Under the strong acid and alkaline condition, the sludge dewatering performance was deteriorated resulting in a small floc and a small volume of supernatant. At low pH, H^+^ on the surface of colloidal sludge increased the electrostatic repulsion between sludge particles, leading to the increase of specific resistance of sludge [15]. When the pH was too high, sludge particle surface would be negatively charged. The drawback was for the flocculant studied that neutralizing the negative charge of the surface of the sludge particles completely was difficult since the repulsion increased between particles would result in an increase in the specific resistance of sludge [16, 17]. As shown in Figure 4, it indicated that the sludge dewatering experiment using P(AM-DAC-BA) was conducted well at the pH of 6–8.

In addition, comparison of dewatering performance between P(AM-DAC-BA) and CPAM was shown in Table 5. As listed in Table 5, DS dewatered by P(AM-DAC-BA) was higher than that dewatered by CPAM. It was evident that the dewatering performance of P(AM-DAC-BA) was better than CPAM on the pH values considered at dosage of 40 mg/L. Dewatering tests demonstrated the superiority of P(AM-DAC-BA) over CPAM.

## 5. Conclusions

Flocculant, P(AM-DAC-BA), was synthesized in this study through UV-initiated polymerization using AM, DAC, and AM as monomers. The best synthetic conditions that were optimized by fitting RSM model while setting the photoinitiator concentration from 0.025 to 0.075 wt%, incident light intensity from 700.0 to 1700.0 *μ*w·cm^2^, and illumination time from 40 to 120 min were given that the cationic degree, photoinitiator concentration, light intensity, and illumination time were 21.94%, 0.6‰, 1491.67 *μ*w·cm^−2^, and 117.89 min, respectively. FTIR provided the support for the formation of P(AM-DAC-BA). In the dewatering experiments of the waste sludge, it presented a good sludge dewatering performance that, with 40 mg·L^−1^ P(AM-DAC-BA) at pH 7, the residual turbidity of supernatant and dry solid content were up to 38 NTU and 28.5%, respectively.

## Figures and Tables

**Table 1:** Experimental ranges and levels of the independent test variables.

Variables	Ranges and levels		
	−1	0	+1
Incident light intensity (*μ*w·cm^−2^) (*X*_1_)	700	1200	1700
Illumination time (min) (*X*_2_)	40	80	120
Photoinitiator concentration (wt.‰) (*X*_3_)	0.25	0.50	0.75

**Table 2:** 3-Factor Box-Behnken design and the value of response function ([*η*]).

Run order	Coded variables			Intrinsic viscosity [*η*] (dL·g^−1^)	
	*X*_1_	*X*_2_	*X*_3_	Actual value	Predicted value
1	0	0	0	1.85	1.86
2	+1	0	1	1.93	1.86
3	−1	0	1	1.45	1.35
4	0	−1	1	1.36	1.42
5	+1	+1	0	2.03	2.00
6	−1	+1	0	1.64	1.63
7	+1	0	−1	0.81	0.90
8	0	0	0	1.85	1.86
9	0	−1	−1	0.71	0.61
10	−1	0	−1	0.90	0.96
11	0	+1	1	1.95	2.04
12	−1	−1	0	1.00	1.02
13	0	0	0	1.80	1.86
14	0	+1	−1	1.57	1.86
15	0	0	0	1.85	1.86
16	+1	−1	0	1.09	1.09
17	0	0	0	1.95	1.86

**Table 3:** ANOVA results for response parameters.

Source	Sum of squares	Degree of freedom	Mean square	*F*-value	*P* value
Regression	3.18	9	0.35	37.33	<0.0001
*X*_1_	0.095	1	0.095	9.99	0.0159
*X*_2_	1.15	1	1.15	121.12	<0.0001
*X*_3_	0.91	1	0.91	96.17	<0.0001
*X*_1_ *X*_2_	0.022	1	0.022	2.37	0.1672
*X*_1_ *X*_3_	0.081	1	0.081	8.57	0.0221
*X*_2_ *X*_3_	0.018	1	0.018	1.92	0.2080
*X*_1_^2^	0.31	1	0.31	33.00	0.0007
*X*_2_^2^	0.092	1	0.092	9.67	0.0171
*X*_3_^3^	0.42	1	0.42	44.09	0.0003
Residual	0.066	7	0.009475		
Lack of fit	0.054	3	0.018	6.04	0.0575
Pure error	0.012	4	0.003		

Total	**3.25**	**16**			
*R* ^2^	0.9796				
*R*_adj_^2^	0.9534				

**Table 4:** Measured and calculated values for confirmation experiments.

Run	Photoinitiator concentration (wt.‰)	Incident light intensity (*μ*w·cm^−2^)	Illumination time (min)	[**η**] (dL·g^−1^)	
				Measured	Calculated
18	0.66	1422.2	102.2	2.15 ± 0.1	2.10
19	0.51	1407.5	101.7	1.99 ± 0.1	2.10
20	0.62	1285.8	88.3	1.89 ± 0.07	2.00

**Table 5:** Comparison of dewatering performance between P(AM-DAC-BA) and CPAM.

DS (%)		pH		
Flocculant	Dosage (mg/L)	4	7	11
P(AM-DAC-BA)	40	16.5	28.4	12.5
CPAM		13.2	26.5	8.1
